# Low muscle quality in Japanese type 2 diabetic patients with visceral fat accumulation

**DOI:** 10.1186/s12933-018-0755-3

**Published:** 2018-08-04

**Authors:** Jun Murai, Hitoshi Nishizawa, Akihito Otsuka, Shiro Fukuda, Yoshimitsu Tanaka, Hirofumi Nagao, Yasuna Sakai, Masahide Suzuki, Shinji Yokota, Hidetoshi Tada, Mayumi Doi, Yuya Fujishima, Shunbun Kita, Tohru Funahashi, Norikazu Maeda, Tadashi Nakamura, Iichiro Shimomura

**Affiliations:** 1 Department of Metabolic Medicine, Graduate School of Medicine, Osaka University, 2-2 B-5, Yamada-oka, Suita, Osaka 565-0871 Japan; 2 Department of Diabetes and Endocrinology, Kawasaki Hospital Kobe, 3-3-1, Higashiyama-Cho, Hyogo-ku, Kobe, 652-0042 Japan; 3 Department of Adipose Management, Graduate School of Medicine, Osaka University, 2-2-B, Yamada-oka, Suita, Osaka 565-0871 Japan; 4 Department of Metabolism and Atherosclerosis, Graduate School of Medicine, Osaka University, 2-2-B, Yamada-oka, Suita, Osaka 565-0871 Japan; 5 Department of Gastroenterology, Kawasaki Hospital Kobe, 3-3-1, Higashiyama-Cho, Hyogo-ku, Kobe, 652-0042 Japan; 6 Department of Laboratory and Physiology, Kawasaki Hospital Kobe, 3-3-1, Higashiyama-Cho, Hyogo-ku, Kobe, 652-0042 Japan

**Keywords:** Type 2 diabetes, Visceral fat accumulation, Sarcopenia, Skeletal muscle index, Grip strength, Muscle quality, Motor nerve conduction velocity, Cardiovascular disease

## Abstract

**Background:**

Although obesity-related type 2 diabetes mellitus (T2DM) and sarcopenia in the elderly have been increasing worldwide, the associations among visceral fat accumulation, skeletal muscle indices (mass, strength, and quality) and cardiovascular diseases in T2DM remain poorly investigated.

**Methods:**

We enrolled 183 Japanese T2DM inpatients (126 men, 57 women; mean age 64.7 ± 12.6 years, ± SD). The estimated-visceral fat area (eVFA) and skeletal muscle mass were measured by each device using bioelectrical impedance analysis method. We also measured grip strength by dynamometer and motor nerve conduction velocity (MCV). We analyzed the difference in skeletal muscle indices between T2DM patients with and without visceral fat accumulation, and examined the impact of skeletal muscle indices on cardiovascular diseases in patients with visceral fat accumulation.

**Results:**

The prevalence of sarcopenia defined by the Consensus of Asian Working Group for Sarcopenia and low skeletal muscle mass were both lower in the visceral fat accumulation (+) group than in (−) group. However, the prevalence of weak hand grip strength was similar in the visceral fat accumulation (−) and (+) groups, indicating that considerable patients with visceral fat accumulation had weak grip strength in spite of fair skeletal muscle mass. Muscle quality [grip strength (kg)/arm muscle mass (kg)] was significantly lower in patients with visceral fat accumulation. Multiple regression analysis identified eVFA, MCV and sex as significant and independent determinants of muscle quality. In visceral fat accumulation (+) group, the patients with low muscle quality had longer duration of diabetes, lower eGFR, higher serum adiponectin, lower MCV and higher prevalence of cardiovascular diseases, compared to the patients with high muscle quality. Finally, sex- and age-adjusted models showed significant association between low muscle quality and cardiovascular diseases in all subjects (odds ratio 2.28, p = 0.012), especially in patients with visceral fat accumulation (odds ratio 2.72, p = 0.018).

**Conclusions:**

T2DM patients with visceral fat accumulation had low muscle quality, and patients with low muscle quality were more affected with cardiovascular diseases.

**Electronic supplementary material:**

The online version of this article (10.1186/s12933-018-0755-3) contains supplementary material, which is available to authorized users.

## Introduction

Obesity-related type 2 diabetes mellitus (T2DM) has been increasing worldwide, especially in Asia [1–4]. Asians are more easily susceptible to T2DM despite of relatively low body mass index (BMI), compared to Caucasians [5]. Body fat distribution varied considerably among individuals. Subcutaneous fat accumulation, e.g. high leg fat mass/total fat mass, was associated with decreased cardiovascular diseases [6]. Irrespective of BMI (< 25 or ≥ 25 kg/m^2^), visceral fat accumulation, which results from dysregulated eating behavior and physical inactivity, leads to type 2 diabetes mellitus, dyslipidemia, hypertension and atherosclerotic cardiovascular diseases, conceptualized the metabolic syndrome [7].

Prevention of sarcopenia, which is defined as reduction in skeletal muscle mass and strength with aging, is important for improvement in quality of life in the elderly [8, 9]. Insulin resistance and diabetes mellitus have been reported to be one of risk factors for sarcopenia and exacerbate its pathology [10–13]. On the other hand, it has been reported that grip strength is independently associated with cardiovascular diseases [14, 15]. Collectively, sarcopenia with obesity-related T2DM may be more susceptible to complications of T2DM, such as cardiovascular diseases.

Taken together, it is clinically important for T2DM patients with visceral fat accumulation to evaluate skeletal muscle mass and strength, in addition to fat distribution. However, data have been scarce on visceral fat accumulation and skeletal muscle in T2DM. The present study was designed to investigate the difference in skeletal muscle indices (mass, grip strength and quality) between T2DM patients with and without visceral fat accumulation. The study was also designed to determine the relation between skeletal muscle indices in T2DM patients with visceral fat accumulation and cardiovascular diseases.

## Methods

### Subjects

The study subjects were consecutive 183 (126 men and 57 women) patients with T2DM admitted to the Division of Diabetes and Endocrinology, Kawasaki Hospital, Kobe, Japan, for poor glycemic control and/or complications, between August 2015 and April 2017. This study was approved by the Human Ethics Committees of Osaka University (no. 15061) and written informed consent was obtained from each subject after explaining the purpose and possible complications of the study. The diagnosis of T2DM was based on the World Health Organization (WHO) National Diabetic Group criteria of 2006 and/or current treatment for diabetes [16]. Patients in whom the visceral fat area (VFA) and/or skeletal muscle mass (see “Clinical examination” below) were not measured were excluded from the study.

### Clinical examination

The duration of diabetes was determined through a medical interview and/or medical records. Height (cm), body weight (kg), waist circumference (WC) at the level of the umbilicus (cm), and grip strength (kg) were measured in the standing position on admission to the hospital. Grip strength was measured using an isokinetic dynamometer (Smedley’s Hand Dynamometer) on the right and left hand and then the average was calculated.

The VFA was estimated by bioelectrical impedance analysis (BIA) method using EW-FA90 (Panasonic Corporation). The VFA measured by the BIA method correlates significantly with that determined by computed tomography, which is the golden standard method for measurement of VFA [17]. In the present study, the muscle masses of the arms and legs, and trunk were measured by In Body 720 (InBody Japan Inc.), which is applied by BIA method. The measured values of muscle mass by the BIA method also correlate with those measured by dual-energy X-ray absorptiometry (DXA) [18]. The arm muscle quality represented the ratio of grip strength to the entire arm muscle mass in kilograms [grip strength (kg)/arm muscle mass (kg)] [19] (measured on both sides and then averages).

Systolic/diastolic blood pressure (BP) was measured with a standard mercury sphygmomanometer on the right and left arms in supine position after at least 10-min rest, and the average was calculated. Venous blood samples were collected in the morning after overnight fasting for measurements of glucose and HbA1c [National Glycohemoglobin Standardization Program (NGSP)], serum C-peptide, alanine transaminase (ALT), uric acid (UA), high-density lipoprotein cholesterol (HDL-C), triglyceride (TG), low-density lipoprotein cholesterol (LDL-C), creatinine, high-sensitivity CRP (hs-CRP), and brain natriuretic peptide (BNP). The estimated glomerular filtration rate (eGFR) was calculated by using the following formula: [eGFR = 194 × (serum creatinine^−1.094^) × (age^−0.287^) × F (male, F = 1; female, F = 0.739)] [20]. Serum levels of total adiponectin were measured by enzyme-linked immunosorbent assay (ELISA) (human adiponectin ELISA kit, Otsuka Pharmaceutical Co. Tokushima, Japan), as reported previously [21].

The maximum intima-media thickness (IMT) of the common carotid artery (CCA) was measured in supine position by echography, as described previously [22, 23]. Arterial stiffness was assessed by measuring brachial-ankle pulse wave velocity (baPWV), ankle-brachial index (ABI), using an automatic wave form analyzer (BP-203RPE III, FUKUDA COLIN Co, Japan).

#### Nerve conduction velocity

To measure motor nerve conduction velocity (MCV), a nerve conduction study (NCS) was performed using standard electrodiagnostic equipment (Neuropack2 MEB-7202, Nihon Koden, Japan) in a temperature controlled room at the Department of Physiology, Kawasaki Hospital. The preparation and equipment settings followed standard protocols [24]. In each patient, the MCV was measured by recording median nerve (MN) activity by supramaximal stimulation of the MN at the proximal site (antecubital fossa) and the distal site (wrist) on each arm [25], and then the average was calculated.

### Definitions

According to the Consensus of Asian Working Group for Sarcopenia (AWGS) [9], weak grip strength was defined as < 26 kg for men and < 18 kg for women. Skeletal muscle index (SMI) was defined as height-adjusted appendicular skeletal muscle mass: muscle mass of the arms and legs/height^2^ (kg/m^2^), based on the definition recommended by AWGS. Low skeletal muscle mass was defined as SMI < 7.0 kg/m^2^ for men and < 5.7 kg/m^2^ for women as measured by BIA. Sarcopenia was defined as weak grip strength and low skeletal muscle mass.

Visceral fat accumulation was defined as estimated VFA (eVFA) of ≥ 100 cm^2^, according to the guideline of Japan Society for the Study of Obesity [26, 27]. Hypertension was defined by systolic BP ≥ 140 mmHg and/or diastolic BP ≥ 90 mmHg. Dyslipidemia was defined as low-density lipoprotein cholesterol (LDL-C) concentration ≥ 140 mg/dl, triglyceride (TG) concentration ≥ 150 mg/dl, and/or high-density lipoprotein cholesterol (HDL-C) concentration < 40 mg/dl. Hyperuricemia was defined as serum uric acid (UA) concentration ≥ 7.0 mg/dl according to the Japanese criteria [28]. Patients were also considered positive for hypertension and/or dyslipidemia and/or hyperuricemia if they received antihypertensive, and/or anti-dyslipidemic, and/or anti-hyperuricemic medications respectively. Fatty liver was defined as presence of liver-to-kidney contrast and/or deep beam attenuation, using abdominal ultrasonography (Aplio MX Diagnostic Ultrasound System SSA780A, Toshiba Medical Systems Inc.) [29]. Cardiovascular disease (CVD) was defined as coronary artery disease (CAD) and/or cerebrovascular disease and/or peripheral arterial disease (PAD). CAD was defined as significant coronary stenosis(es) by coronary angiography or computed tomography, and/or positive ischemia by stress myocardial scintigraphy. Cerebrovascular disease was defined as history of stroke by a medical interview and/or old cerebral infarction by magnetic resonance imaging (MRI). PAD was defined as ankle-brachial index (ABI) ≤ 0.9 or performance of a revascularization procedure or amputation of lower extremity because of PAD [30, 31].

#### Statistical analysis

Data are presented as mean ± SD or median (interquartile range). In all cases, probability (*p*) value of < 0.05 was considered statistically significant. The unpaired *t* test was used to determine differences in various parameters between two groups. Frequencies were compared between two groups by Fisher’s exact test. The correlations between muscle quality and other parameters were first analyzed by simple linear regression analysis and then by multiple regression analysis (Tables 2, 3). To clarify clinical features of patients with visceral fat accumulation with low muscle quality, the subjects were classified into four groups based on visceral fat accumulation and muscle quality; the low group < 10.3 (kg/kg), which is the median value of muscle quality, and the high group ≥ 10.3 (kg/kg) (Table 4). The associations between muscle quality/grip strength and prevalence of CVD were assessed by logistic regression analysis (Table 5, Additional file 1: Tables S2, S3). Models that examined CVD as the outcome included adjustments for the following covariates: sex, age, sex and age. All analyses were performed with the JMP Pro 10.0.2 software for Windows (SAS Institute, Cary, NC).

## Results

### Prevalence of low skeletal muscle mass, weak grip strength, and sarcopenia

We enrolled 183 Japanese patients in this study [126 men and 57 women; age 64.7 ± 12.6 years; range, 33-88 years, BMI 25.3 ± 4.7 kg/m^2^, WC; males 93.1 ± 13.0 cm, females 95.3 ± 12.8 cm, eVFA; males 145.2 ± 60.0 cm^2^, females 121.0 ± 49.2 cm^2^] (Table 1). Among the total, 57 cases (31%) had low skeletal muscle mass, as measured by BIA (SMI, men: < 7.0, women: < 5.7 kg/m^2^), and 104 cases (57%) had weak grip strength (men: < 26 kg, women: < 18 kg). Accordingly, 41 (22%) T2DM patients were diagnosed with sarcopenia. In sex-specific analyses, 40 cases (32%) in men and 17 cases (30%) in women had low skeletal muscle mass, and 60 cases (48%) in men and 44 cases (77%) in women had weak grip strength. Then, 26 cases (21%) in men and 15 cases (26%) in women were diagnosed with sarcopenia.

**Table 1:** Clinical characteristics of study subjects

n (males/females)	183 (126/57)
Age (years)	64.7 ± 12.6
Duration of diabetes (years)	9 (3–21)
BMI (kg/m^2^)	25.3 ± 4.7
WC (cm)	93.8 ± 12.9
Males	93.1 ± 13.0
Females	95.2 ± 12.8
eVFA (cm^2^)	137.7 ± 57.8
Males	145.2 ± 60.0
Females	121.0 ± 49.2
Grip strength (kg)	23.5 ± 9.5
Males	27.5 ± 8.1
Females	14.8 ± 6.1
Skeletal mass index (kg/m^2^)	7.1 ± 1.2
Males	7.5 ± 1.0
Females	6.1 ± 1.1
Systolic BP (mmHg)	131.7 ± 19.3
Diastolic BP (mmHg)	76.4 ± 12.0
HbA1c (%)	9.5 ± 2.1
Serum C-peptide (ng/ml)	1.8 ± 1.3
ALT (U/l)	31.2 ± 31.1
UA (mg/dl)	5.3 ± 1.6
eGFR (ml/min)	67.3 ± 28.3
HDL-C (mg/dl)	46.1 ± 16.8
TG (mg/dl)	148 (105–217)
LDL-C (mg/dl)	106.5 ± 36.8
hs-CRP (mg/dl)	0.12 (0.06–0.35)
Adiponectin (μg/ml)	7.3 (4.6–11.3)
BNP (pg/ml)	15.7 (7.7–35.3)
CCA max IMT (mm)	0.9 ± 0.5
PWV (m/s)	1810 ± 379
MCV (m/s)	49.5 ± 4.4
Hypertension	68%
Hyperuricemia	26%
Dyslipidemia	86%
Fatty liver	58% (105/180)
Cardiovascular disease	34%

Data are presented as mean ± SD, or median (interquartile range), or frequency % (number of subjects)

*BMI* body mass index, *WC* waist circumference, *eVFA* estimated visceral fat area, *BP* blood pressure, *ALT* alanine transaminase, *UA* uric acid, *eGFR* estimated glomerular filtration rate, *HDL-C* HDL cholesterol, *TG* triglyceride, *LDL-C* LDL cholesterol, *hs-CRP* high sensitive C-reactive protein, *BNP* brain natriuretic peptide, *CCA* common carotid artery, *IMT* intima-media thickness, *PWV* pulse wave velocity, *MCV* motor nerve conduction velocity

**Fig. 1:**
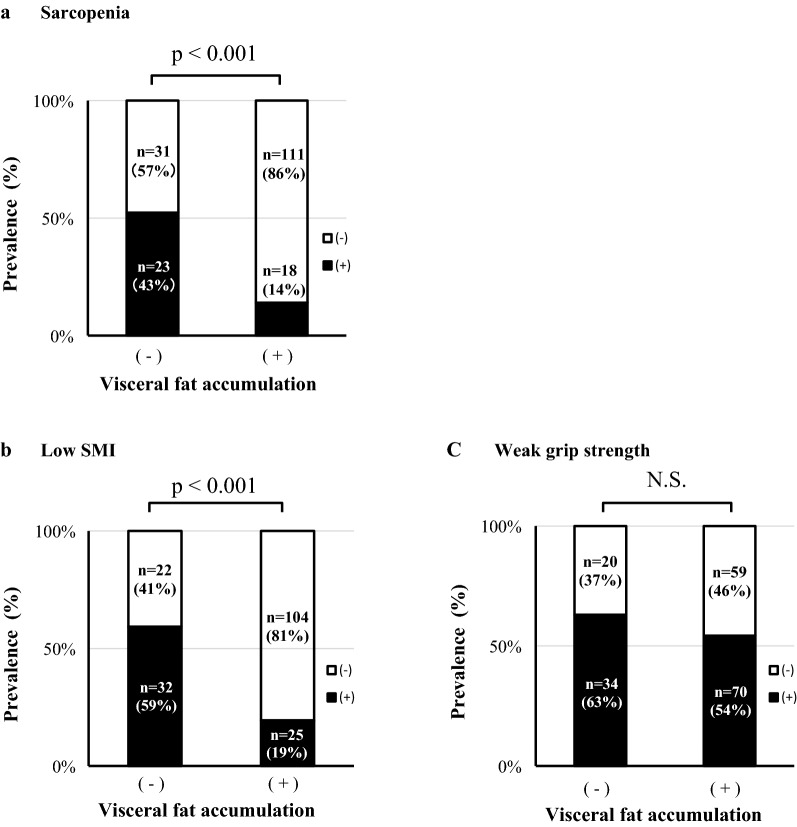
Visceral fat accumulation and prevalence of sarcopenia, low skeletal muscle mass, and weak grip strength. The subjects were divided into two groups: visceral fat accumulation (−) group and visceral fat accumulation (+) group. Visceral fat accumulation was defined as eVFA ≥ 100 cm^2^. *p* value by Fischer’s exact test. *SMI* skeletal muscle index = appendicular skeletal muscle mass/height^2^ (kg/m^2^). Definition of sarcopenia, low skeletal muscle mass and weak grip strength are described in “Methods” section. *NS* not significant

Next, we divided the patients into two groups; patients with and those without visceral fat accumulation, and examined the prevalence of sarcopenia and its related factors (Fig. 1). Sarcopenia was observed in 14% (18/129) of visceral accumulation (+) group and 43% (23/54) of visceral accumulation (−) group (p < 0.001) (Fig. 1a). Low skeletal muscle mass diagnosed by low SMI was observed in 19% (25/129) of visceral accumulation (+) group and in 59% (32/54) of (−) group (p < 0.001) (Fig. 1b). The prevalence of sarcopenia and low skeletal muscle mass were both lesser in (+) group than in (−) group (Fig. 1a, b). On the other hand, the prevalence of weak hand grip strength was not different between the two groups [63% (34/54) in (−) group, and 54% (70/129) in (+) group] (Fig. 1c). In sex-specific analyses, almost the same data were observed both in men and in women (Additional file 1: Figure S1). These data suggest that patients with visceral fat accumulation tended to have weak hand grip strength in spite of fair skeletal muscle mass.

**Fig. 2:**
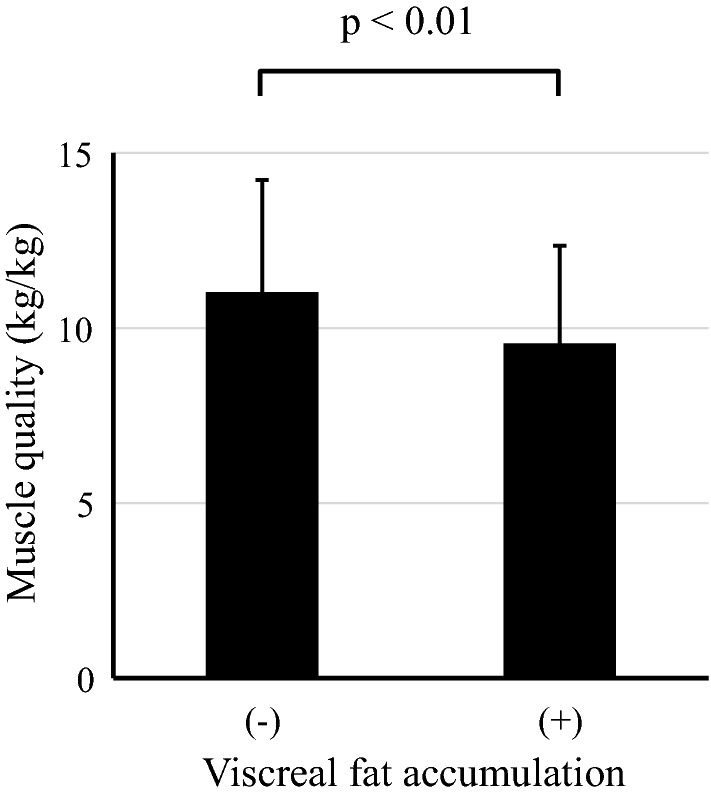
Visceral fat accumulation and muscle quality (grip strength/arm skeletal muscle mass). The subjects were divided into two groups: visceral fat accumulation (−) group and visceral fat accumulation (+) group. Visceral fat accumulation was defined as estimated visceral fat area ≥ 100 cm^2^. Muscle quality was defined as muscle strength divided by skeletal muscle mass [grip strength (kg)/arm muscle mass (kg)]. *p* value by unpaired t test

Then, we focused on muscle quality, which is defined as muscle strength divided by skeletal muscle mass [19]. As shown in Fig. 2, the muscle quality was significantly lower in patients with visceral fat accumulation than those without. In sex-specific analyses, the muscle quality was significantly lower in patients with visceral fat accumulation both in men and in women (Additional file 1: Figure S2). Additional file 1: Table S1 provides more details on skeletal muscle indices. Although all indices of muscle mass were clearly larger in patients with visceral fat accumulation, grip strength was not significantly different in both males and females.

### Clinical parameters associated with muscle quality

Simple linear regression analysis showed significant relationships between muscle quality and age, duration of diabetes, BMI, eVFA, eGFR, LDL-C, CCA max IMT, PWV, and MCV (Table 2). Sex difference was observed in muscle quality (men 10.6 ± 2.7 vs women 8.6 ± 3.1, p < 0.001). Then, multiple regression analysis which included age, sex, duration of diabetes, eVFA, eGFR, LDL-C, CCA max IMT, MCV identified sex, eVFA and MCV as significant explanatory variables associated with muscle quality (Table 3).

**Table 2:** Simple regression analyses of the factors associated with muscle quality

	Simple linear regression model	
	r	*p* value
Age (years)	− 0.185	*0.012*
Duration of diabetes (years)	− 0.191	*0.018*
BMI (kg/m^2^)	− 0.350	*<* *0.001*
eVFA (cm^2^)	− 0.266	*<* *0.001*
Systolic BP (mmHg)	− 0.062	0.407
Diastolic BP (mmHg)	0.085	0.255
HbA1c (%)	0.103	0.173
Serum C-peptide (ng/ml)	− 0.126	0.091
ALT (U/l)	0.062	0.404
UA (mg/dl)	− 0.002	0.981
eGFR (ml/min)	0.245	*0.001*
HDL-C (mg/dl)	0.129	0.081
TG (mg/dl)	0.120	0.105
LDL-C (mg/dl)	0.204	*0.006*
hs-CRP (mg/dl)	− 0.097	0.205
Adiponectin (μg/ml)	− 0.071	0.341
BNP (pg/ml)	0.088	0.242
CCA max IMT (mm)	− 0.159	*0.044*
PWV (m/s)	− 0.196	*0.009*
MCV (m/s)	0.303	*<* *0.001*

**Table 3:** Multiple regression analysis of the factors associated with muscle quality

	Multiple regression model	
	Std β	*p* value
Age (years)	− 0.048	0.599
Sex	0.405	*<* *0.001*
Duration of diabetes (years)	− 0.094	0.270
eVFA (cm^2^)	− 0.348	*<* *0.001*
eGFR (ml/min)	− 0.085	0.320
LDL-C (mg/dl)	0.081	0.270
CCA max IMT (mm)	− 0.116	0.120
PWV (m/s)	− 0.127	0.140
MCV (m/s)	0.301	*<* *0.001*

### Clinical features of T2DM patients with visceral fat accumulation and low muscle quality

Based on the above results, we focused on the clinical features of patients with both visceral fat accumulation and low muscle quality. The subjects were classified into four groups based on the eVFA and muscle quality (Table 4). In visceral fat accumulation (+) group, the patients with low muscle quality had longer duration of diabetes, lower eGFR, higher serum adiponectin, and lower MCV, compared to the patients with high muscle quality. No significant differences were observed in the prevalence of hypertension, hyperuricemia, dyslipidemia and fatty liver between the two groups. Interestingly, the prevalence of atherosclerotic cardiovascular diseases was clearly higher in the low muscle quality group with visceral fat accumulation; 10 cases (18%) of the high muscle quality group, versus 30 cases (41%) of the low muscle quality group of subjects with visceral fat accumulation (p = 0.007) (Table 4).

**Table 4:** Clinical features of type 2 diabetic patients with visceral fat accumulation and low muscle quality

	Visceral fat accumulation (−) group			Visceral fat accumulation (+) group		
	Muscle quality		*p* value	Muscle quality		*p* value
	High group	Low group		High group	Low group	
n (males/females)	36 (25/11)	18 (8/10)	0.087^a^	56 (46/10)	73 (47/26)	*0.030* ^a^
Age (years)	65.5 ± 11.4	74.3 ± 7.7	0.005	61.4 ± 13.3	64.4 ± 12.5	0.192
Duration of diabetes (years)	5 (0–30)	20 (8–29)	0.344	5 (1–13)	12 (6–21)	*0.007*
BMI (kg/m^2^)	20.0 ± 2.4	21.5 ± 2.7	*0.040*	26.5 ± 3.8	27.9 ± 4.0	*0.042*
WC (cm)	79.3 ± 6.7	83.3 ± 8.5	0.066	97.8 ± 10.3	100.5 ± 10.5	0.125
eVFA (cm^2^)	63.3 ± 21.7	75.1 ± 21.5	0.067	164.8 ± 39.7	168.9 ± 40.0	0.565
Grip strength (kg)	24.8 ± 7.7	13.4 ± 5.4	*<* *0.001*	30.9 ± 8.0	20.0 ± 7.8	*<* *0.001*
Skeletal mass index (kg/m^2^)	6.3 ± 0.9	6.0 ± 0.9	0.213	7.4 ± 0.9	7.4 ± 1.2	0.945
Systolic BP (mmHg)	131.7 ± 24.1	140.0 ± 22.2	0.224	130.2 ± 15.3	130.9 ± 18.6	0.831
Diastolic BP (mmHg)	77.0 ± 11.5	72.9 ± 9.4	0.196	77.8 ± 11.0	76.0 ± 13.4	0.414
HbA1c (%)	9.6 ± 2.2	10.1 ± 3.5	0.605	9.9 ± 1.9	9.2 ± 1.7	*0.032*
Serum C-peptide (ng/ml)	1.2 ± 0.8	1.9 ± 1.8	0.058	2.0 ± 1.4	1.9 ± 1.2	0.679
ALT (U/l)	29.2 ± 37.6	21.1 ± 12.8	0.380	35.2 ± 26.3	31.5 ± 33.8	0.580
UA (mg/dl)	4.5 ± 1.6	5.0 ± 1.6	0.355	5.6 ± 1.3	5.4 ± 1.6	0.394
eGFR (ml/min)	76.8 ± 31.9	57.0 ± 26.5	*0.027*	72.9 ± 26.0	61.1 ± 26.6	*0.016*
HDL-C (mg/dl)	57.3 ± 23.1	47.9 ± 17.4	0.135	45.0 ± 13.3	41.1 ± 12.5	0.090
TG (mg/dl)	123 (82–173)	116 (82–167)	0.890	187 (127–267)	152 (113–213)	0.077
LDL-C (mg/dl)	101.7 ± 33.9	91.1 ± 38.5	0.306	119.4 ± 38.3	102.7 ± 34.4	*0.011*
hs-CRP (mg/dl)	0.1 (0.0–0.2)	0.2 (0.1–0.6)	0.153	0.1 (0.1–0.4)	0.1 (0.1–0.4)	0.301
Adiponectin (μg/ml)	11.3 (7.3–16.9)	8.2 (6.5–15.7)	0.878	5.3 (3.7–8.2)	6.6 (4.8–10.6)	*0.009*
BNP (pg/ml)	22.4 (15.0–59.5)	28.0 (15.1–49.9)	0.302	10.7 (4.9–21.7)	16.4 (8.1–29.8)	0.726
CCA max IMT (mm)	0.8 ± 0.4	1.3 ± 0.8	*0.013*	0.9 ± 0.4	0.9 ± 0.4	0.505
PWV (m/s)	1791 ± 463	2051 ± 370	0.054	1744 ± 329	1814 ± 358	0.262
MCV (m/s)	50.2 ± 3.7	48.3 ± 4.1	0.100	50.8 ± 3.9	48.5 ± 4.9	*0.003*
Hypertension	58%	72%	0.382^a^	64%	75%	0.180^a^
Hyperuricemia	17%	17%	1.000^a^	30%	30%	1.000^a^
Dyslipidemia	72%	89%	0.298^a^	91%	89%	0.775^a^
Fatty liver	33%	28%	0.764^a^	76% (41/54)	65% (47/72)	0.241^a^
Cardiovascular disease	33%	61%	0.080^a^	18%	41%	*0.007* ^a^

Data are expressed as mean ± SD, or median (interquartile range), or frequency % (number of subjects). Unpaired t test was used between low muscle quality group vs high muscle quality group

^a^Fisher’s exact test (low muscle quality group vs high muscle quality group)

### Association of low muscle quality with atherosclerotic cardiovascular diseases

We next investigated the impact of lower muscle quality on cardiovascular diseases using logistic regression analysis (Table 5). In the sex- and age-adjusted models, lower muscle quality was significantly associated with cardiovascular diseases in overall subjects (odds ratio 2.28, p = 0.012). However, there was no significant difference in the associations between lower muscle quality and cardiovascular diseases in sex- and age-adjusted models subjects without visceral fat accumulation (p = 0.134). Interestingly, in patients with visceral fat accumulation, the association of lower muscle quality with cardiovascular diseases was significant as higher odds ratio in sex- and age- adjusted models (odds ratio 2.72, p = 0.018). In male subjects only, significant associations of lower muscle quality with cardiovascular disease were observed in overall patients and patients with visceral fat accumulation (odds ratio 2.59, p = 0.020, odds ratio 4.60, p = 0.006, respectively), but not in patients without visceral fat accumulation, in the age-adjusted models (Additional file 1: Table S2). Finally, we examined the association of hand grip strength with cardiovascular diseases. There were significant associations of weak grip strength with cardiovascular diseases in overall patients with/without visceral fat accumulation (Additional file 1: Table S3).

**Table 5:** Association of low muscle quality for cardiovascular disease in type 2 diabetic patients

	Odds ratio	*p* value
All		
Not adjusted	2.61 (1.40–4.97)	*0.003*
Sex adjusted	2.40 (1.27–4.62)	*0.007*
Age adjusted	2.42 (1.28–4.66)	*0.006*
Sex, age adjusted	2.28 (1.20–4.42)	*0.012*
eVFA < 100 cm^2^		
Not adjusted	3.14 (0.99–10.61)	0.052
Sex adjusted	3.39 (1.03–12.27)	*0.045*
Age adjusted	2.43 (0.70–8.81)	0.162
Sex, age adjusted	2.64 (0.74–10.05)	0.134
eVFA ≥ 100 cm^2^		
Not adjusted	3.21 (1.44–7.64)	*0.004*
Sex adjusted	2.83 (1.24–6.83)	*0.013*
Age adjusted	3.05 (1.36–7.31)	*0.007*
Sex, age adjusted	2.72 (1.19–6.61)	*0.018*

## Discussion

### Sarcopenia and skeletal muscle indices in type 2 diabetic patients with visceral fat accumulation

In the present study, sarcopenia was detected in 22% of the hospitalized patients with T2DM (age 64.7 ± 12.6 years; range 33–88 years). Since the reported prevalence of sarcopenia is 1–29% in community-dwelling populations [32] and 7.5–8.2% in Japanese population aged over 60 or 65 years [33, 34], the prevalence of sarcopenia in our study of T2DM is relatively higher than in the general population. These results confirm the previous findings that T2DM patients are susceptible to sarcopenia [35, 36].

First, we focused on visceral fat accumulation in T2DM. The prevalence of sarcopenia and low skeletal muscle mass were both lower in patients with visceral fat accumulation (Fig. 1a, b), probably reflecting relative larger amount of muscle mass due to overweight. On the other hand, the prevalence of weak hand grip strength was not influenced by the amount of visceral fat (Fig. 1c), indicating that considerable patients with visceral fat accumulation had weak hand grip strength despite fair skeletal muscle mass. It was recently reported that the patients with decreased grip strength and abdominal obesity were at higher risk of worsening disability [37].

### Low muscle quality in type 2 diabetic patients with visceral fat accumulation, and its association with cardiovascular diseases

Then, we focused on muscle quality. As shown in Fig. 2, muscle quality of patients with visceral fat accumulation was significantly lower than in those without. Interestingly, multiple regression analysis identified eVFA and MCV as significant determinants of muscle quality (Table 3). Previous studies reported lower muscle quality in T2DM, compared with subjects without diabetes, and was associated with the duration of diabetes [19]. Collectively, these results suggest that visceral fat accumulation and/or neuropathy per se can be associated with muscle quality in T2DM. Our results also showed that low muscle quality had significant impact on cardiovascular diseases in both the sex- and age-adjusted models (Table 5 and Additional file 1: Table S2), suggesting that T2DM patients with visceral fat accumulation and low muscle quality could be at higher risk of atherosclerotic cardiovascular diseases.

The reported mechanism(s) of decreased muscle strength in diabetes include insulin resistance [12], insulin signaling [38], neuropathy [39], and hyperglycemia [19]. Since MCV correlated significantly with muscle quality (Tables 2, 3), it is possible that neuropathic changes could be involved in the decrease of muscle strength through motoneurons dysfunction.

The exact etiology of cardiovascular diseases and decreased muscle quality in T2DM with visceral fat accumulation is unknown. Dysregulation of adipocytokines/adipokines, such as hypoadiponectinemia, has been reported previously [40], and the high levels of inflammatory cytokines in the accumulated visceral fat could result in systemic low-grade inflammation, insulin resistance, microalbuminuria [41, 42], arterial stiffness [43], and atherosclerotic cardiovascular diseases [40, 44]. In fact, hypercytokinemia, including high serum levels of tumor necrosis factor-α and interleukin 6 (IL-6), has been reported to be associated with both muscle mass and muscle strength in the elderly [45, 46]. Moreover, it was reported that subjects with abdominal obesity and sarcopenia had high plasma levels of IL-6 [47]. Accumulation of visceral fat had a significant impact on the gene expression profile in peripheral blood cells [48], and also on muscle-derived bioactive molecules called myokines [47, 49]. Systemic inflammation and dysregulation of adipocytokines in visceral fat accumulation might be associated with decreased muscle quality and atherosclerosis. We have recently reported that adiponectin is associated with skeletal muscle via T-cadherin [50]. Although it is well known that adiponectin acts as an insulin-sensitizer in skeletal muscles [51], its effects on skeletal muscle mass and strength remain elusive.

It has been reported that grip strength is inversely associated with mortality [14, 52]. In the present study, grip strength correlated significantly with cardiovascular diseases (Additional file 1: Table S3). However, 20% (n = 16, 54.7 ± 11.6 years) of patients without weak grip strength (n = 79, 58.0 ± 12.3 years) had lower muscle quality [< 10.3 (kg/kg)]. Since considerable number of overweight subjects have relatively larger muscle and increased total fat mass, it may not be appropriate to use absolute values of muscle strength in the analysis. Accordingly, we need to evaluate muscle strength per muscle mass, i.e., muscle quality, particularly in subjects with visceral fat accumulation. On the other hand, in patients without visceral fat accumulation, grip strength correlated significantly with cardiovascular diseases (Additional file 1: Table S3), suggesting that it is important to evaluate the absolute value of grip strength especially in patients free of visceral fat accumulation. In male patients, lower muscle quality was also associated with cardiovascular diseases (Additional file 1: Table S2). However, due to the limited number, analyses of female patients could not be performed.

Recently, it was reported that skeletal muscle indices were positively correlated with C-peptide, which showed endogenous insulin reserve [53], and dipeptidyl peptidase 4 inhibitors attenuated the decline of skeletal muscle in patients with T2DM [54]. Taken together, the present results demonstrated the importance of evaluating muscle quality in type 2 diabetic patients with visceral fat accumulation for potential risk assessment for atherosclerosis, suggesting that physicians might be better to consider selection of anti-diabetic agent from view point of the effect on skeletal muscle and support for patients in modifying their exercise habit.

Our study was cross-sectional in design conducted in a single medical facility and could thus only examine associations among visceral fat accumulation, muscle quality, and cardiovascular diseases. For any causal relationship, larger cohort and prospective studies are needed. In the present study, the methods of measurement for VFA and skeletal muscle mass were BIA methods (EW-FA90 and In Body 720, respectively) as alternatives of CT method and DXA method, respectively.

## Conclusion

Our study demonstrated that patients with T2DM and visceral fat accumulation had lower muscle quality, and that patients with low muscle quality were more affected with cardiovascular diseases.

## Additional file

**Additional file 1: Figure S1.** Visceral fat accumulation and prevalence of sarcopenia, low skeletal muscle mass, and weak grip strength (men, women). The subjects were divided into two groups: visceral fat accumulation (−) group and visceral fat accumulation (+) group. p value by Fischer’s exact test. NS: not significant. **Figure S2.** Visceral fat accumulation and muscle quality (men, women). The male and female subjects were divided into two groups: visceral fat accumulation (−) group and visceral fat accumulation (+) group. *p* value by unpaired t test. **Table S1.** Skeletal muscle indices of type 2 diabetic patients with visceral fat accumulation. **Table S2.** Association of low muscle quality for cardiovascular disease in type 2 diabetic patients (men). **Table S3.** Association of weak grip strength for cardiovascular disease in type 2 diabetic patients.

## Abbreviations

ALT: alanine transaminase
BMI: body mass index
BP: blood pressure
BNP: brain natriuretic peptide
CCA: common carotid artery
eVFA: estimated visceral fat area
eGFR: estimated glomerular filtration rate
HDL-C: HDL cholesterol
hs-CRP: high sensitive C-reactive protein
IL-6: interleukin 6
IMT: intima-media thickness
LDL-C: LDL cholesterol
MCV: motor nerve conduction velocity
PWV: pulse wave velocity
SMI: skeletal mass index
TG: triglyceride
UA: uric acid
VFA: visceral fat area
WC: waist circumference
