# Supplementary material for: Low muscle quality in Japanese type 2 diabetic patients with visceral fat accumulation
Source: Cardiovasc Diabetol. 2018 Aug 4;17:112. doi: 10.1186/s12933-018-0755-3 (PMC6076400; doi:10.1186/s12933-018-0755-3)

**Additional file 1: Table S1. Skeletal muscle indices of type 2 diabetic patients with visceral fat accumulation.**

|  | All | Visceral fat accumulation | | *p* value |
| --- | --- | --- | --- | --- |
|  |  | (-) group | (+) group |  |
| n (males/females) | 126 / 57 | 33 / 21 | 93 / 36 | 0.163^+^ |
| Age (years) | 64.7±12.6 | 68.4±11.1 | 63.1±12.9 | **0.009** |
| Duration of diabetes (years) | 9 (3-21) | 10 (1-30) | 9 (3-20) | **0.045** |
| BMI (kg/m^2^) | 25.3±4.7 | 20.5±2.6 | 27.3±4.0 | **<0.001** |
| WC (cm) | 93.8±12.9 | 80.6±7.5 | 99.3±10.5 | **<0.001** |
| Males | 93.1±13.0 | 79.1±6.4 | 98.1±10.9 | **<0.001** |
| Females | 95.2±12.8 | 82.9±8.5 | 102.4±8.7 | **<0.001** |
| eVFA (cm^2^) | 137.7±57.8 | 67.2±22.2 | 167.2±39.8 | **<0.001** |
| Grip strength (kg) | 23.5±9.5 | 21.0±8.8 | 24.6±9.6 | **0.018** |
| Males | 27.5±8.1 | 25.3±7.7 | 28.2±8.1 | 0.077 |
| Females | 14.8±6.1 | 14.1±5.5 | 15.3±6.4 | 0.483 |
| Skeletal mass index (kg/m^2^) | 7.1±1.2 | 6.2±0.9 | 7.4±1.1 | **<0.001** |
| Males | 7.5±1.0 | 6.6±0.6 | 7.8±1.0 | **<0.001** |
| Females | 6.1±1.1 | 5.4±0.8 | 6.5±1.0 | **<0.001** |
| Arm muscle mass (kg) | 4.7±1.4 | 3.7±1.0 | 5.1±1.3 | **<0.001** |
| Males | 5.3±1.2 | 4.3±0.8 | 5.6±1.2 | **<0.001** |
| Females | 3.5±0.9 | 2.9±0.6 | 3.9±0.9 | **<0.001** |
| Leg muscle mass (kg) | 11.1±3.8 | 9.5±3.2 | 11.8±3.8 | **<0.001** |
| Males | 12.4±3.6 | 11.0±3.0 | 13.0±3.6 | **0.006** |
| Females | 8.3±2.6 | 7.2±2.1 | 8.9±2.6 | **0.016** |
| Trunk muscle mass (kg) | 20.1±4.3 | 17.1±3.2 | 21.4±4.1 | **<0.001** |
| Males | 21.8±3.7 | 18.9±2.5 | 22.9±3.5 | **<0.001** |
| Females | 16.3±2.9 | 14.2±1.8 | 17.5±2.9 | **<0.001** |

Unpaired t test (visceral fat accumulation (-) group vs (+) group)

^+^: Fisher’s exact test (visceral fat accumulation (-) group vs (+) group)

**Additional file 1: Table S2. Association of low muscle quality for cardiovascular disease in type 2 diabetic patients (men).**

|  | Odds ratio | *p* value |
| --- | --- | --- |
| All men |  |  |
| not adjusted | 2.80 (1.27-6.43) | **0.010** |
| age adjusted | 2.59 (1.16-6.01) | **0.020** |
| Men with eVFA <100 cm^2^ |  |  |
| not adjusted | 4.00 (0.83-23.22) | 0.084 |
| age adjusted | 3.43 (0.67-20.65) | 0.139 |
| Men with eVFA ≥100 cm^2^ |  |  |
| not adjusted | 4.75 (1.59-17.66) | **0.004** |
| age adjusted | 4.60 (1.52-17.31) | **0.006** |

**Additional file 1: Table S3. Association of weak grip strength for cardiovascular disease in type 2 diabetic patients.**

|  | Odds ratio | *p* value |
| --- | --- | --- |
| All |  |  |
| not adjusted | 4.70 (2.37-9.86) | **<0.001** |
| sex adjusted | 4.33 (2.14-9.23) | **<0.001** |
| age adjusted | 4.11 (1.93-9.21) | **<0.001** |
| sex, age adjusted | 3.83 (1.78-8.69) | **<0.001** |
| eVFA<100 cm^2^ |  |  |
| not adjusted | 5.07 (1.50-20.66) | **0.008** |
| sex adjusted | 5.53 (1.57-23.63) | **0.007** |
| age adjusted | 4.19 (1.06-20.02) | **0.041** |
| sex, age adjusted | 4.44 (1.11-21.43) | **0.035** |
| eVFA ≥ 100 cm^2^ |  |  |
| not adjusted | 4.42 (1.95-10.86) | **<0.001** |
| sex adjusted | 3.76 (1.61-9.46) | **0.002** |
| age adjusted | 4.11 (1.67-10.91) | **0.002** |
| sex, age adjusted | 3.47 (1.37-9.41) | **0.008** |

**Additional file 1: Figure S1. Visceral fat accumulation and prevalence of sarcopenia, low skeletal muscle mass, and weak grip strength (men, women).**

The subjects were divided into two groups: visceral fat accumulation (-) group and visceral fat accumulation (+) group. *P* value by Fischer's exact test. N.S.: not significant.


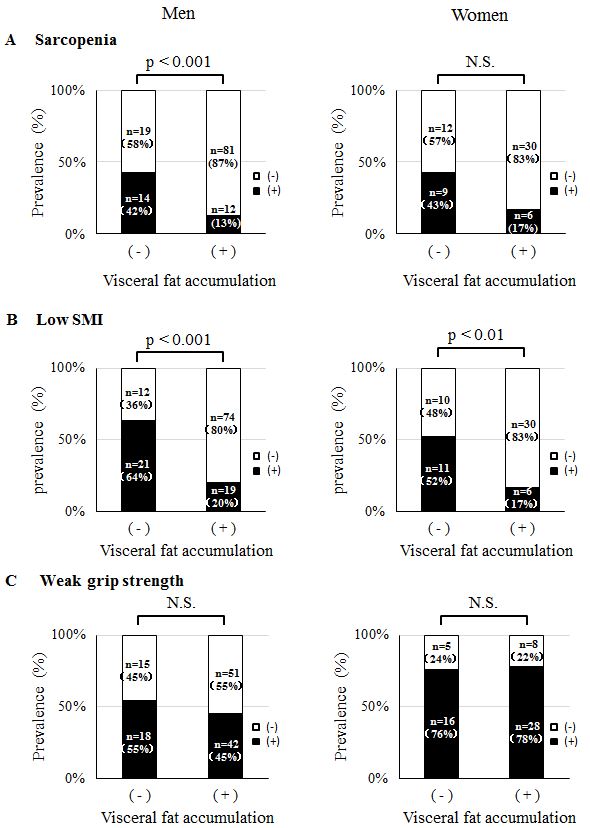


**Additional file 1: Figure S2. Visceral fat accumulation and muscle quality (men, women).**

The male and female subjects were divided into two groups: visceral fat accumulation (-) group and visceral fat accumulation (+) group. *P* value by unpaired t test.


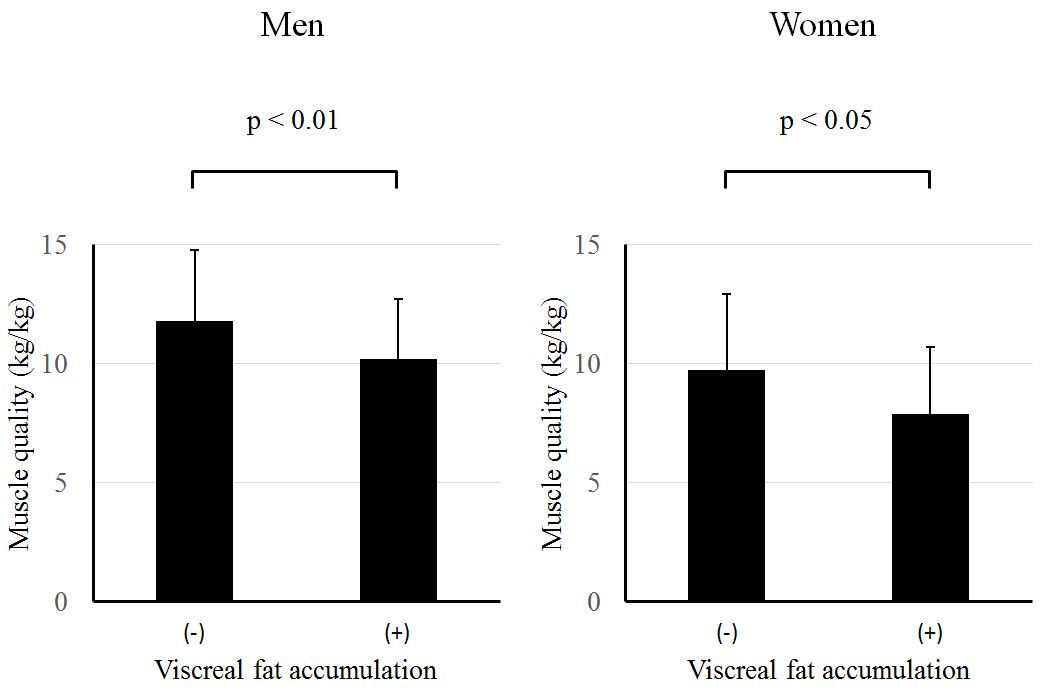

Supplement: Supplementary file 1 — Additional file 1: Figure S1. Visceral fat accumulation and prevalence of sarcopenia, low skeletal muscle mass, and weak grip strength (men, women). The subjects were divided into two groups: visceral fat accumulation (−) group and visceral fat accumulation (+) group. p value by Fischer’s exact test. NS: not significant. Figure S2. Visceral fat accumulation and muscle quality (men, women). The male and female subjects were divided into two groups: visceral fat accumulation (−) group and visceral fat accumulation (+) group. p value by unpaired t test. Table S1. Skeletal muscle indices of type 2 diabetic patients with visceral fat accumulation. Table S2. Association of low muscle quality for cardiovascular disease in type 2 diabetic patients (men). Table S3. Association of weak grip strength for cardiovascular disease in type 2 diabetic patients. [file 12933_2018_755_MOESM1_ESM.docx]
